# The mediating role of job burnout in the relationship between career plateau and turnover intention among nurses

**DOI:** 10.1038/s41598-025-19518-1

**Published:** 2025-10-10

**Authors:** Shaghyegh Arzani, Mohammad Amerzadeh, Ahad Alizadeh, Saeideh Moosavi, Rohollah Kalhor

**Affiliations:** 1 Student Research Committee, School of Public Health, Qazvin University of Medical Sciences, Qazvin, Iran; 2 Non-communicable Diseases Research Center, Research Institute for Prevention of Non-Communicable Diseases, Qazvin University of Medical Sciences, Qazvin, Iran; 3 Medical Microbiology Research Center, Qazvin University of Medical Sciences, Qazvin, Iran

**Keywords:** Career plateau, Job burnout, Nurses, Turnover intention, Health care, Medical research

## Abstract

High turnover rates can hinder hospitals and the healthcare system from achieving their goals of providing high-quality medical services. Consequently, turnover intention (TI) has emerged as a critical issue within healthcare environments, adversely affecting the quality of care delivered to patients. This study investigates the relationship between career plateau (CP) and TI among nurses, with job burnout (JB) serving as a mediating factor. This cross-sectional study was conducted from February to November 2021. The population comprised 1,289 nurses from Qazvin University of Medical Sciences (QUMS) teaching hospitals. According to Morgan’s sample size table, the required sample size was determined to be 297 nurses. A stratified sampling method was employed to ensure a representative sample. We utilized standardized questionnaires, including Milliman’s CP Scale, Maslach’s JB Inventory, and Kim-Leong’s TI Scale. Statistical analyses were performed using Structural Equation Modeling (SEM), t-tests, ANOVA, Pearson correlation tests, and regression analyses to explore the relationships among the variables. The results were analyzed using R software version 4.1.1. The means and standard deviations for the study variables were as follows: CP (M = 38.08, SD = 5.75), JB (M = 65.99, SD = 16.68), and TI (M = 8.20, SD = 3.31). The results indicated that CP has an indirect relationship with TI, mediated by job burnout. Significant positive correlations were observed among the variables: JB with TI, JB with CP, and CP with TI (*p* < 0.05), both content plateau and hierarchical plateau scores were higher in female nurses. Additionally, mean scores for job burnout, emotional exhaustion, depersonalization, and TI were also higher among female nurses. Our findings demonstrate that CP significantly influences JB and TI among nurses. The implications of TI are substantial, as they incur high costs related to recruitment, training, and employee retention. Therefore, healthcare managers should adopt human resource management strategies aimed at alleviating workload and working hours, promoting physical and mental health, reducing stress levels, and enhancing communication skills. Implementing these strategies can improve job satisfaction among nurses and ultimately reduce burnout and TI.

## Background

Effectively maintaining human resources remains a significant challenge for organizations, as the loss of skilled employees not only depletes institutional expertise but can also substantially hinder overall performance^1,2^. In this context, *turnover intention* (TI)—defined as the likelihood of an employee leaving within a specified timeframe^3^.

The workforce shortage crisis in the healthcare system significantly impacts disease prevention and health promotion in many countries^4,5^. While some studies attribute declining care quality primarily to increasing demand for emergency services and high workloads, others emphasize the role of inadequate staffing ratios^6^. However, methodological differences—such as reliance on cross-sectional surveys versus longitudinal workforce data—may partly explain inconsistencies in reported effect sizes^7,8^. Numerous studies have explored the influence of human resources on care quality and the staffing levels of nurses in hospitals^9^. As a result, both nurses and doctors are continuously subjected to these challenges^10,11^.

The healthcare sector is a vital area for sustainable development due to its direct impact on human health. Nurses often experience fatigue from dealing with numerous challenges and job-related stress, which can lead to TI^12^. Freneau et al. originally defined a career plateau (CP) as “a point in the career where the probability of promotion is very low.“^13^. Traditionally, CP is described as a stage in a job where advancement appears unlikely^14^. Effective management of CP is essential to prevent employee dissatisfaction^15^.

Research on CPs has identified two primary types: hierarchical plateaus and career content plateaus^16^. A hierarchical plateau occurs when there are no opportunities for promotion within an organization, while a career content plateau refers to lateral stagnation, where individuals do not take on additional responsibilities or find their jobs sufficiently challenging. Burnout is not merely a symptom of job stress; rather, it is a consequence of unmanaged job stress, characterized by emotional exhaustion, depersonalization, and a perceived lack of personal achievement^16,17^.

Evidence indicates that CP can lead to negative outcomes, including decreased job satisfaction, reduced enjoyment at work, and increased TI. Factors such as high workload, lack of autonomy, and poor work-life balance can contribute to the development of CP^18^. Conversely, studies have shown that employees who experience higher levels of job compatibility tend to have lower instances of CP. When individuals find a better fit with their job and organization, it can reduce the likelihood of experiencing a CP^19^.

TI is a critical variable for organizations due to its detrimental effects and financial implications, including the costs associated with recruiting, replacing, and training new staff, as well as the time wasted in the process^20^. Furthermore, nurses, as the largest group of healthcare providers, contribute significantly to inefficiencies in care delivery when they express high levels of TI. This issue hampers hospitals and healthcare systems in their efforts to provide quality medical services^21^. Substantial evidence suggests that CP is an increasing concern that is associated with negative outcomes, including heightened TI^18^.Therefore, the main hypothesis and aim of this study posits that there is a relationship between CP and TI, mediated by job burnout (JB), among nurses working in hospitals in Qazvin.

**Fig. 1:**
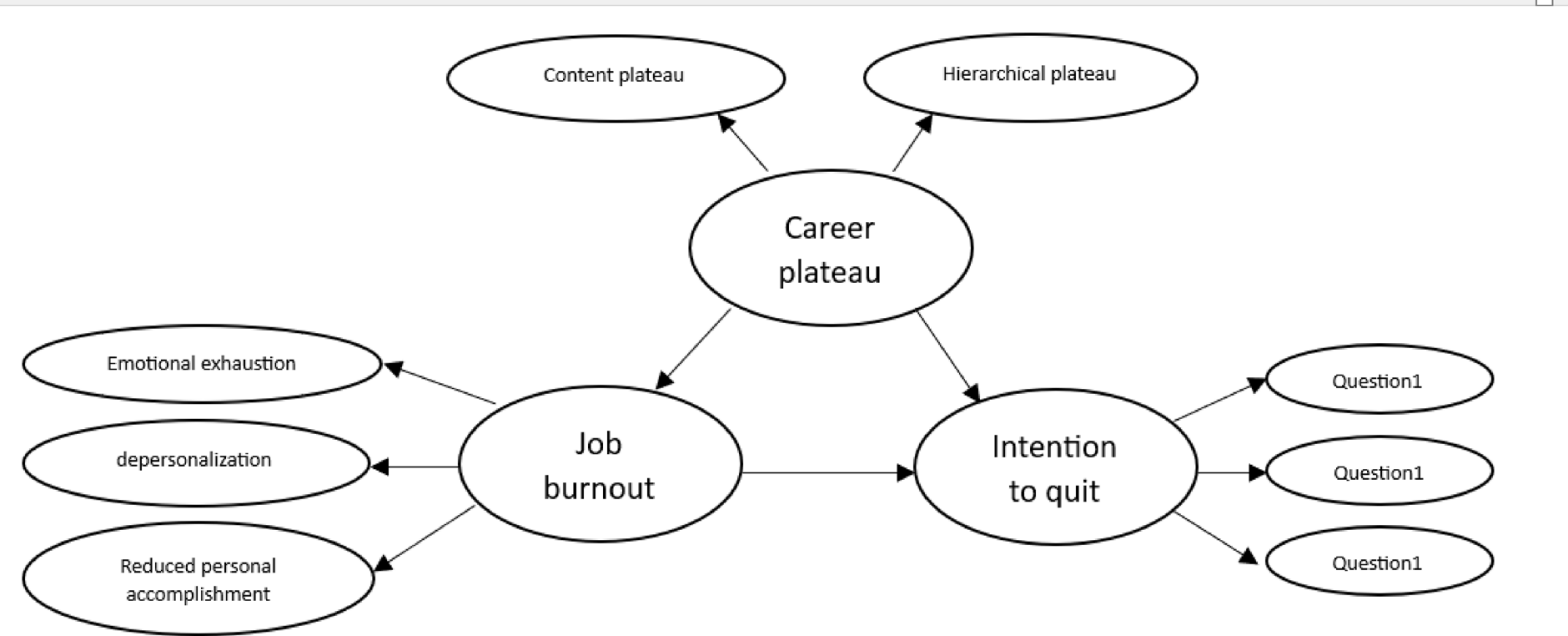
conceptual model of research.

In many countries today, high levels of job stress among professionals have emerged as a significant social and economic challenge, contributing to a decline in quality of life and an increase in TI^19^. This issue is commonly referred to as JB. In particular, JB among nurses is a pressing concern within the nursing community and the healthcare system, as it can lead to higher TI^19^. Consequently, this study aims to investigate the relationship between CP and nurses’ TI, with JB serving as a mediating factor, in teaching hospitals affiliated with QUMS. Also, determining the status of CP, JB and TI in nurses in Qazvin are another aims and hypothesis in this study (Fig. 1).

While the mediated relationship between CP, JB, and TI has been explored in other contexts, this study provides theoretical value by validating these constructs in a healthcare setting in Iran, where organizational and cultural dynamics may influence these relationships differently. Additionally, by using Structural Equation Modeling (SEM) and exploring subgroup differences by gender, education level, and work shifts, the study extends prior models and confirms that contextual variables must be considered in understanding the impact of career stagnation on burnout and retention. These findings can inform localized HR practices and burnout prevention policies in public hospitals.

## Methods

### Study design and setting

This descriptive-analytical study was conducted cross-sectionally from February to November 2021. The study population consisted of nurses affiliated with QUMS. This study design was chosen to investigate the relationships between the independent variable, CP, and several dependent variables, including TI and JB.

## Sample size and sampling

The total number of nurses in the population was 1,289. Based on Morgan’s table, the required sample size was determined to be 297. We employed stratified sampling, where the number of participants in each stratum was established, and systematic random sampling was used to select individuals within each stratum.The minimum number of samples for structural equation modeling studies is 5 samples and a maximum of 15 samples per item^22^. Considering the 46 items in this study, the number of numerical samples was between 230 and 690. Due to the limited statistical population, Morgan’s table was used to determine the number of samples, and finally 297 samples and 342 samples were considered for this study, including 15% attrition. All participating nurses had more than one year of work experience.

The sample sizes from Rajaei, Kausar, Bu Ali, Quds, Velayat, and 22 Bahman hospitals were 59, 36, 84, 41, 66, and 11, respectively. A total of 342 samples were initially considered for this research, accounting for a 10% dropout rate. After adjustments, the final sample sizes were 70, 41, 95, 48, 75, and 13 for Rajaei, Kausar, Bu Ali, Quds, Velayat, and 22 Bahman hospitals, respectively. We included nurses who volunteered to participate and had at least one year of work experience. Data were collected by visiting the hospitals under study. In this way, when the researcher visited the workplace of the individuals in question, after obtaining permission and providing explanations about the objectives of the study and how to complete the questionnaire, the printed sheets of the questionnaire were provided to the respondents for completion. Then, the questionnaires were collected and after ensuring that all questions were answered carefully(method-com5), they were entered into the software for analysis that It takes 1 month. Nurses with less than one year of experience and those who declined to participate for any reason were excluded from the study. The response rate for the study was 97%.

## Data collection tools

The questionnaire used for data collection consisted of four sections. The first section gathered demographic information, including age, gender, marital status, education, work experience, and organizational position. The second, third, and fourth sections included Milliman’s CP questionnaire, Kim-Leung’s TI questionnaire, and Maslach’s JB questionnaire, respectively^22–24^. The JB questionnaire consists of 22 items that measure three dimensions: emotional exhaustion, depersonalization, and a lack of personal accomplishment within the context of professional activity. It is primarily designed to assess burnout among professional groups such as nurses and teachers. The scoring system utilizes a 7-point Likert scale. The validity and reliability of the JB questionnaire were confirmed by Ahmadi and Filian, while the TI questionnaire was validated in Barani’s study^25–27^. Milliman developed the CP questionnaire, which examines CP from various dimensions, including structural plateau and content plateau. This questionnaire is comprised of 12 items and is scored on a 5-point Likert scale. The validity and reliability of Milliman’s CP questionnaire were reported as optimal in studies by Zardashtian (0.84) and Jung Un (0.84)and Asmaa Elwan Mohammed Hassan^28,29^. The TI questionnaire consists of three items that assess an individual’s willingness to leave their job, also using a 5-point Likert scale, where higher scores indicate a greater intention to leave. The reliability of this questionnaire was reported at 0.745 in Barani’s study using Cronbach’s alpha and at 0.86 in Bamari’s study^26^.

### Data analysis

In this study, we employed various statistical techniques to analyze the data. The primary methodology used was SEM, which enabled us to examine the relationships between our dependent and independent variables. It shows how these variables are related and how an independent variable can explain the dependent variables. Additionally, we utilized t-tests, ANOVA, Pearson correlation tests, and regression analyses to further investigate these relationships. We conducted all analyses using the R programming language and the lavaan package.

Multivariate analysis is one of the most robust methods for analyzing data in behavioral and social science research. Given the complex nature of the issues studied, which involve multiple variables, a two-variable analysis is often insufficient. Multivariate analysis encompasses a range of techniques that allow for the simultaneous examination of K independent variables and N dependent variables. Structural equation modeling is a key approach for analyzing covariance structures and causal relationships in complex data.

Considering the necessary and sufficient sample size calculated based on the Morgan table, using this analysis SEM provided reliable results of means, correlations between variables, standard coefficients, and standard error.

In addition to SEM, we assessed construct validity, including both convergent and discriminant validity. Discriminant validity was tested using the Fornell-Larcker criterion and the Heterotrait-Monotrait (HTMT) ratio to confirm that CP, JB, and TI are empirically distinct constructs.

#### Ethical approval and consent to participate

This article is based on a portion of a dissertation (Ethics code IR.QUMS.REC.1400.336) completed for a Master’s degree. All protocols were approved by the Ethical Committee of Qazvin University of Medical Sciences. The questionnaires were collected anonymously, and confidentiality was maintained throughout the study. The results will be shared with hospital managers. All methods were conducted in accordance with relevant guidelines and regulations. Participants, or their legal guardians, were provided with an information sheet that assured them of their anonymity, their right to withdraw from the study, and the confidentiality of their information. They were also informed about the purpose of the study and asked to provide their informed consent.

## Results

### Demographic findings

The response rate for the study was 97%. The findings revealed that 249 participants (84.29%) were women, and 187 participants (62.96%) were married (Table 1). The average age of the participants was 32.14 years, with an average service experience of 8.44 years and management experience of 6 years. Participants reported an average of 52.85 working hours per week (Table 2).

In terms of education, 90.06% held a bachelor’s degree. Regarding job positions, 94.36% were nurses, 2.42% were head nurses, 1.81% were supervisors, and 0.91% were matrons. Additionally, 92.33% of participants worked rotating shifts.

**Table 1:** Average of Participants.

	Count	Percentage
Man	52	71/15
Women	280	29/84
Total	332	100

**Table 2:** Average participant characteristics.

Characteristic	Average
Age	32.14 years
Service Experience	8.44 years
Management Experience	6 years
Working Hours per Week	52.85 h

## Subgroup analyses

The results of the main variables, analyzed by gender, indicated that female nurses had higher mean scores for CP and its dimensions, specifically content plateau and hierarchical plateau. Additionally, the mean scores for job burnout, emotional exhaustion, and depersonalization were also higher among female nurses.

However, there was a notable reduction in personal accomplishment among male nurses. Additionally, the average TI was higher in female nurses. A significant difference was found between the mean scores of depersonalization and TI based on participants’ gender (*p* < 0.05). No significant differences were observed in the other variables (*p* > 0.05).

The means of the research variables based on the level of education revealed that nurses with bachelor’s degrees had higher averages for CP (38.12), hierarchical plateau (19.07), JB (69.09), and depersonalization (10.72). In contrast, nurses with postgraduate education exhibited higher averages for content plateau (20.15), emotional exhaustion (28.46), and reduced personal accomplishment (31.21). TI was highest among nurses with an associate degree, averaging 8.80, compared to other educational levels. However, there were no significant differences between the average scores of nurses’ education levels and the research variables (*p* > 0.05).‏‎‎‏.

The means of the research variables based on work shifts indicated that nurses with fixed work shifts had higher average scores for content plateau, reduced personal accomplishment, and TI. A significant difference was observed in the average depersonalization scores between nurses with fixed shifts and those with rotating shifts (*p* < 0.05). However, no significant differences were found for the other variables related to shift work (*p* > 0.05). The results indicated no significant relationship between CP, JB, and TI with the age of nurses. Additionally, a positive correlation was found between CP and length of service. In contrast, JB and TI exhibited a negative correlation with length of service. However, there was no significant correlation between CP, job burnout, and TI related to length of service (*p* > 0.05).

The results indicated a correlation between the research variables and working hours; however, there was no significant relationship between CP, JB, and TI with working hours (*p* > 0.05).

### Main variable

The frequency distribution of research variables presented in Table 3 indicates that nurses’ CP, JB, and TI are at moderate levels.

**Table 3:** Distribution of the main research variable.

	Average	Standard deviation	The range of points
CP	38.08	5.74	(00/41 − 00/35)
Hierarchical plateau	96/18	94/3	
Content plateau	13/1	16/3	
JB	99/68	65/16	(75/80 − 00/57)
Emotional exhaustion	28/28	54/10	
Depersonalization	52/10	00/7	
Reduced personal accomplishment	95/29	46/6	
TI	20/8	31/3	(00/11–00/6)

SEM was employed to assess the model fit. Table 4 demonstrates that several goodness-of-fit indices fall within the defined range.Since our goal in this research is not prediction but merely the effect and relationship between variables, goodness of fit indices are not of great value to us.

**Table 4:** Redefined conceptual model fit indices.

Index name	Limit	The amount
$\:\frac{{\varvec{\chi\:}}^{2}}{\varvec{d}\varvec{f}}$	**Less than 3**	**3/0**
**GFI**	**Above 0.9**	**0/94**
**RMSEA**	**Less than 0.08**	**0/90**
**CFI**	**Above 0.9**	**0/92**
**NFI**	**Above 0.9**	**0/90**

SEM was employed to assess model fit. Table 4 demonstrates that several goodness-of-fit indices fall within the acceptable range. Although the RMSEA value was slightly above the conventional cutoff (0.09 vs. <0.08), it is still considered acceptable for models with complex structures or large sample sizes, especially when other fit indices (CFI = 0.92, GFI = 0.94, NFI = 0.90) indicate good fit. As our goal was to examine the relationships among variables rather than prediction, slight deviations from strict cutoffs are unlikely to compromise the interpretability of results.

### SEM results

**Table 5:** The relationship between CP and TI.

		Confidence interval		0.95%		
**Variables**	**Lower limit**	**Upper limit**	**STD)** standard coefficient)		**SE (standard error)**	**P-value**
**Direct relationship**	**−0/58**	**0/005**	**−0/51**		**0/15**	**0/05**
**Indirect relationship**	**0/01**	**0/71**	**0/73**		**0/15**	**0/008**
**general relationship**	**0/02**	**0/22**	**0/22**		**0/05**	**0/01**

As a main result in this research, According Table 3,career plateau has an indirect relationship with the intention to quit (*P* < 0.05). And for every one-unit increase in plateauing, the intention to quit increases indirectly by 41% through the mediation of job burnout. Also, in the overall relationship for every one-unit increase in plateauing, the tendency to quit increases by 12.

Table 5 indicates a significant relationship between CP and JB (*p* < 0.05). The findings show that JB increases by 0.31 units for each one-unit increase in CP. Furthermore, for each unit increase in job burnout, TI) rises by 0.13 units (*p* < 0.05). Additionally, a one-unit increase in CP results in a 0.29 unit increase in TI. Notably, the effect of JB on TI is nearly twice as significant as the effects of CP on JB and CP on TI. (Table 5)

**Table 6:** Regression weights in structural equation model parameters.

**Variables**	**lower limit**	Confidence interval		0.95%		**P-value**
		**upper limit**	**STD (standard coefficient**)		**SE (standard error)**	
**CP- JB**	**1/90**	**4/30**	**0/69**		**0/61**	**< 001/0**
**DB-TI**	**−0/07**	**0/19**	**1/07**		**0/03**	**< 001/0**
**CP-TI**	**−0/58**	**< 001/0**	**−0/52**		**0/15**	**0/05**

The results showed that there is a positive and significant correlation between CP and TI (Table 6).

**Table 7:** Correlation of variables.

Cp with JB	< 0/001	0/40
Cp with TI	0/005	0/16
JB with TI	< 0/001	0/46

We used ANOVA to examine the relationships between the research variables, employment status, and education level. The results showed no significant relationship between employment status and the research variables (*p* > 0.05). However, there was a significant relationship between CP, content plateau, and JB with education level (*p* < 0.05).

**Fig. 2:**
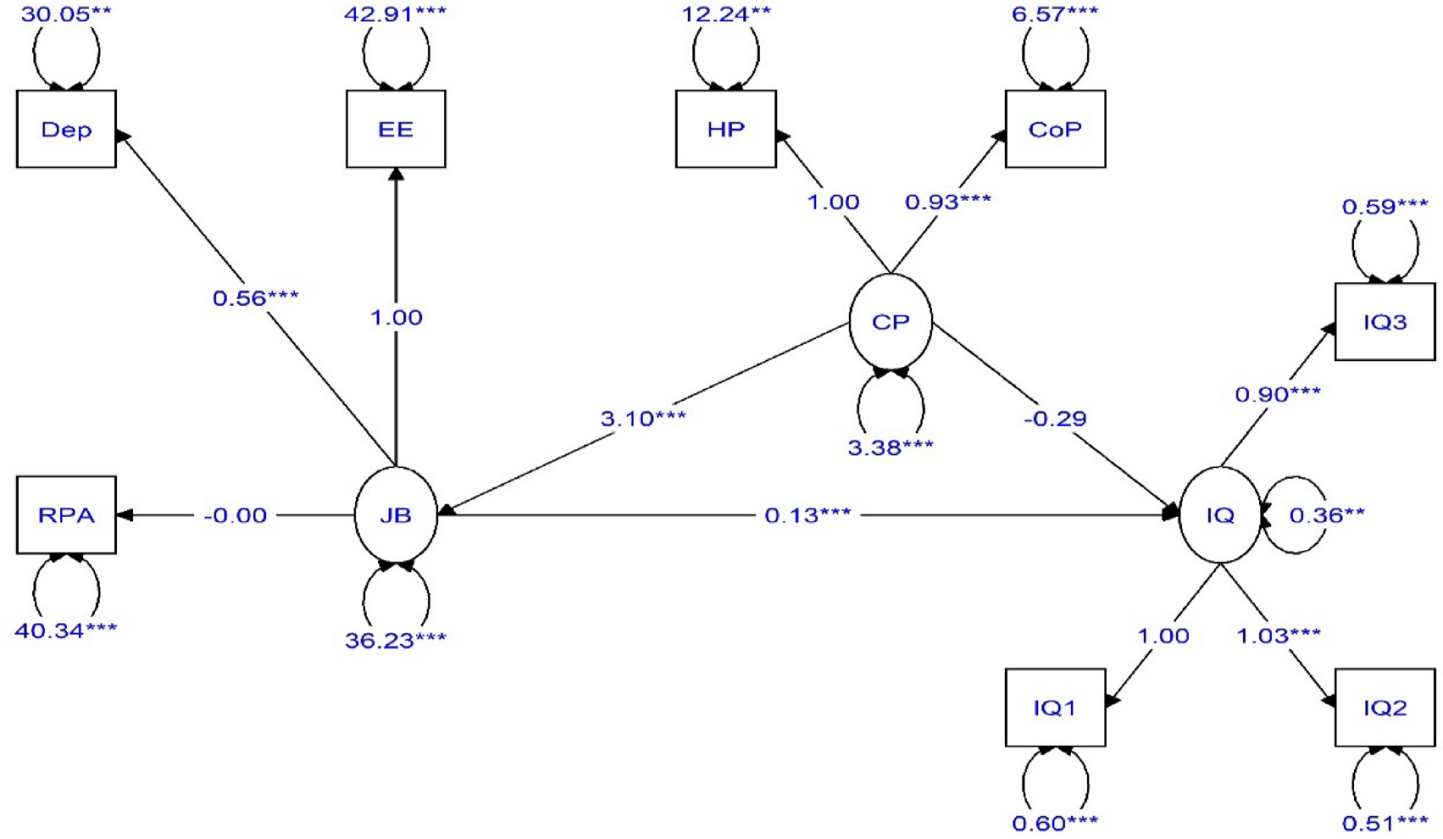
Estimation of the research model with non-standard coefficients.

**Fig. 3:**
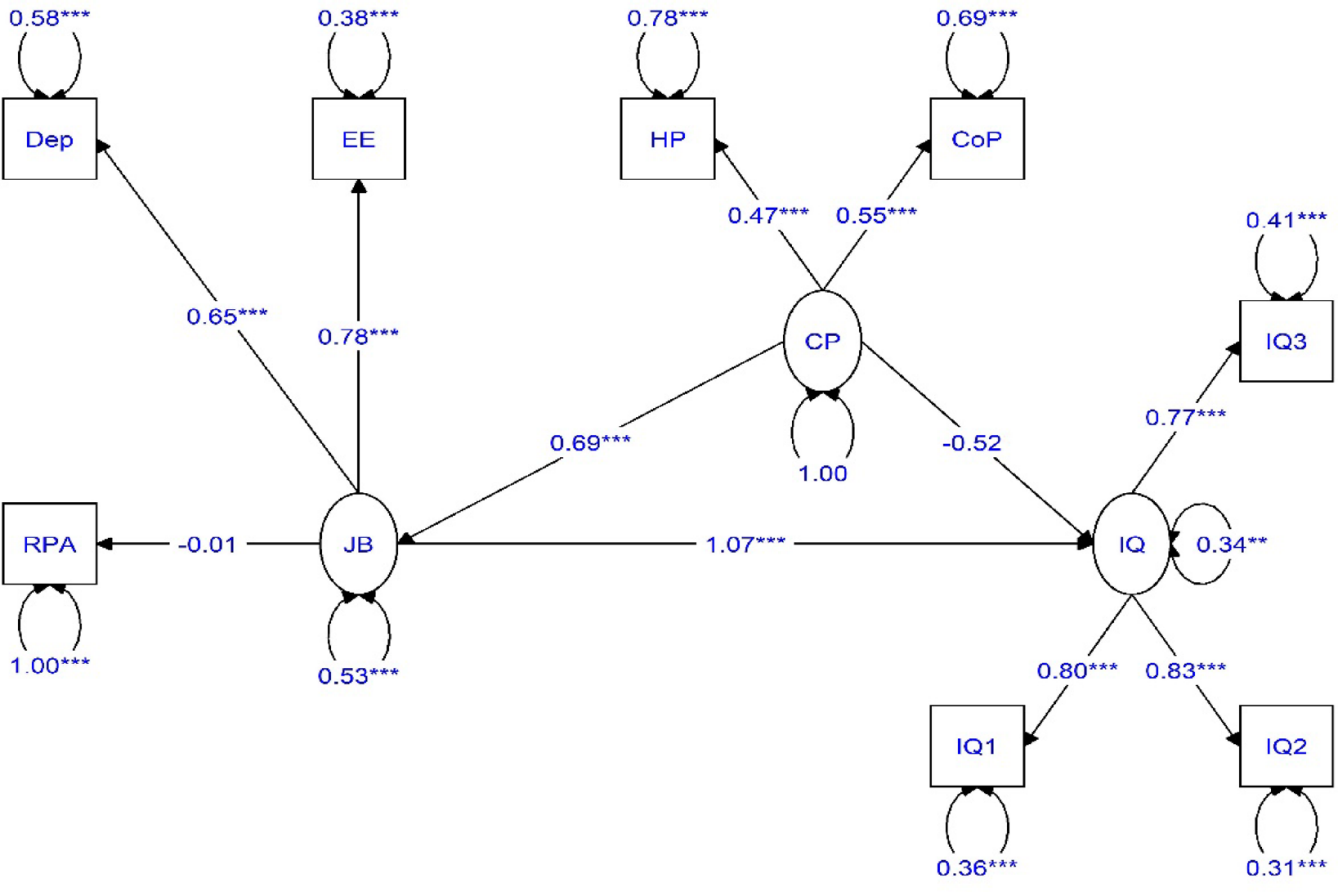
Estimation of the research model with standard coefficients.

Figures 2 and 3 illustrate the path coefficients among the model variables, reflecting the research assumptions. The non-standard path coefficients range between positive infinity and negative infinity. A positive coefficient indicates a positive relationship between the research variables. Two asterisks denote significance at the 0.01 level, while three asterisks indicate significance at the 0.001 le.

## Discussion

This study investigated the impact of CP on TI, mediated by JB, among nurses working at QUMS. Our findings revealed that the mean and standard deviation of CP among nurses were 38.08 and 5.74, respectively, indicating a moderate level of CP. This result aligns with previous studies by Kim Y-m, Nantsupawat D. Liu, Mehrban, Ebrahimi Endrami, and Hydari^30–37^.

In reviewing other studies, Mehraban reported that there is an average CP among employees of the Tabriz City Education Department^32^. In another study, the mean and standard deviation of CP were found to be 36.65 and 6.43, respectively, indicating a moderate level of CP^31^. Liu found that CP among healthcare providers in Hauling, China, was 61.8%, indicating a high prevalence of JB. The most influential factors identified were marital status, weekly working hours, monthly income, and years of service^33^.

Ebrahimi’s study indicated that CP in Iranian university libraries was average. Hydari concluded that the CP level at Tehran University was higher than at other universities, with a greater incidence of structural plateau in the humanities department compared to the primary science department. Results showed a high rate of CP across these three universities^34,36^.Kim Y-M found that as CP decreases among nurses, TI also decreases^31^. Nantsupawat concluded that there is a positive relationship between JB and TI in nurses^30^.

In our study, the mean and standard deviation of JB were 68.99 and 16.65, respectively, which aligns with findings from the studies of Najafi Pour Zatsagh, Sullivan, Amiri, Lazalouya, Al-Ravished, Tanunu, Mahdinjad, Mohammadnia, and Emadian^37–45^. In our studythere was a significant difference in the mean depersonalization score between individuals with fixed shifts and rotating shifts.Najafi Pour’s study reported that the mean and standard deviation of JB among teachers and employees in Zanjan City were 3.57 and 0.85, respectively^37^. Sullivan concluded that JB increased among nurses with the onset of the COVID-19 pandemic, reporting emotional exhaustion at 68% and depersonalization at 88.3%. This study identified factors such as job stress, insufficient staffing, and low income as contributing to JB^38^. Amiri found that out of 384 participants, 232 (61%) exhibited JB syndrome, with a higher prevalence among married individuals compared to singles. Additionally, the stress caused by COVID-19 was linked to an increase in JB^40^.

Another study revealed that 49% of nurses exhibited signs of JB, indicating that its prevalence among nurses is higher than among other groups providing medical services^39^. A similar study found that the prevalence of JB among doctors was 57.7%. Factors such as gender, working in multiple hospitals, long working hours, night shifts, lack of access to personal protective equipment, and a positive COVID-19 test result were significantly associated with JB^42^.

Tanuno’s research reported the mean and standard deviation of nurses’ JB dimensions, revealing emotional exhaustion at 11.5 and 11.4, depersonalization at 4.4 and 5.0, and reduced personal accomplishment at 40.1 and 8.5. These findings indicate a very high prevalence of reduced personal accomplishment among nurses. Mahdi’s research found that the mean and standard deviation of JB among teachers were 47.76 and 15.25, respectively^41^. Mohammadnia reported that the mean and standard deviation of JB in women were 52.2 and 4.6, respectively, while for men, they were 5.48 and 5.8. This suggests that JB is more prevalent in women than in men^42^.

Emadiyan’s study found that the mean and standard deviation of JB among teachers before the intervention were 88.60 and 11.53, respectively. After the intervention, these values changed to 69.87 and 6.72, indicating that job empowerment effectively reduces JB among teachers^45^. These findings suggest that JB is a prevalent issue among both nurses and teachers, influenced by factors such as job stress, inadequate staffing, and low income. Implementing job empowerment strategies and reducing stress may help alleviate JB.

Furthermore, the finding that nurses working fixed shifts reported significantly higher levels of depersonalization (*p* < 0.05) deserves special attention. Depersonalization is a key dimension of job burnout and is often associated with emotional detachment and reduced empathy toward patients. This suggests that fixed shifts, despite their predictability, may lead to psychological distancing or monotony that exacerbates burnout. Given the moderate levels of career plateau reported in our study, it is plausible that fixed shifts contribute to feelings of stagnation and role monotony—hallmarks of content plateau—which in turn heighten depersonalization. This highlights the need to consider shift type as a contributing factor to both CP and JB, especially in rigid work environments like hospitals.

In our study, the mean and standard deviation of TI were 8.20 and 3.31, respectively, indicating a moderate level of TI, consistent with findings from Haji, Schug, Taghavi, and Barani^20,26,46,47^. Haji’s study identified a positive and significant relationship between job stress and TI, with an average TI rate reported^20^. Katrina found that 18.9% of nurses experienced TI^46^. Taghavi’s research demonstrated a positive and significant relationship between moral tension and TI^47^. Barani reported that the mean and standard deviation of TI among teachers were 8.22 and 3.13, respectively, indicating that destructive leadership and organizational injustice are positively and significantly related to TI^26^. These findings suggest that factors such as job stress, moral tension, destructive leadership, and organizational injustice are potential predictors of TI among nurses and teachers.

In this study, we found that CP had a direct and significant effect on TI and also influenced TI indirectly through the mediation of JB. The simultaneous significance of both direct and indirect paths indicates that job burnout partially mediates the relationship between CP and TI. These findings highlight the importance of addressing both structural career challenges and burnout symptoms independently when designing retention strategies for nurses. Zardashtian’s study corroborated these findings, showing that CP impacted nurses’ TI both directly and indirectly through JB^28^. Conversely, Hassan Zadeh’s research indicated a positive and significant effect of TI on JB, which contrasts with our results^48^. Additionally, Ahmadi’s study found that the effect of CP on TI was not statistically significant, also contradicting our findings^25^. Dashtgerd and Qhadiri reported no significant relationship between TI and JB, which further differs from our results^49,50^.

The increase in TI incurs substantial costs for organizations, as they invest heavily in recruitment, training, development, and retention of employees. These investments are at risk of being wasted when employees leave. Therefore, it is crucial for managers to focus on reducing TI. In this context, CP should be recognized as a significant factor; organizations must address and minimize this phenomenon among employees. As many organizations are moving toward flatter structures, the likelihood of CP becomes inevitable. Consequently, organizations should seek ways to enhance employee motivation and effectiveness. Implementing human resource management strategies will help create conditions that allow employees to achieve a successful CP.

Given that the findings of this research indicate that job plateau variables significantly impact JB and TI among nurses, it is crucial to address these issues to maintain the quality of care provided. To prevent JB among nurses, particularly in the area of reduced personal accomplishment, strategies aimed at decreasing burnout and TI should be prioritized within the nursing community.

Based on the findings of this study, which indicated a moderate level of CP, job burnout, and TI among nurses in Qazvin, it can be concluded that the presence of a CP negatively affects nurses’ JB and may lead to decreased attention to their responsibilities. Therefore, it is essential for health managers to focus on improving this situation.

As a recommendation, a more comprehensive study should be conducted with a larger sample size across multiple provinces and research centers. Additionally, the impact of nurses’ CP and burnout on their job satisfaction and stress levels should be further explored.

## Conclusion

This study found that CP is positively associated with JB and TI, with TI posing risks to care quality. To address these issues, managers should adopt human resource strategies such as reducing workloads, enhancing well-being programs, fostering autonomy, improving communication, and clarifying job roles. Nursing education can also prepare students for workplace realities to improve career alignment. A committed workforce aligned with organizational values benefits both service quality and staff retention. Implementing the suggested measures can improve job satisfaction, reduce burnout, and lower TI among nurses. Future research should use longitudinal designs, include larger and more diverse healthcare staff across institutions, and examine cultural differences to better understand and address CP, JB, and TI in nursing.

## Data Availability

The datasets used and/or analyzed during the current study available from the corresponding author ‎on reasonable request. The entire dataset is in Farsi language. The Data can be available in English ‎language for the readers and make available from the corresponding author on reasonable request.
